# Larvicidal, Ovicidal, Synergistic, and Repellent Activities of *Sophora alopecuroides* and Its Dominant Constituents Against *Aedes albopictus*

**DOI:** 10.3390/insects11040246

**Published:** 2020-04-15

**Authors:** Rana Fartab Shoukat, Muhammad Shakeel, Syed Arif Hussain Rizvi, Junaid Zafar, Yuxin Zhang, Shoaib Freed, Xiaoxia Xu, Fengliang Jin

**Affiliations:** 1 College of Agriculture, South China Agricultural University, Laboratory of Bio-Pesticide Creation and Application of Guangdong Province, Guangzhou 510642, China; ranafartab@gmail.com (R.F.S.); faizaneabiwaqas@scau.edu.cn (M.S.); arifrizvi30@yahoo.com (S.A.H.R.); jz_jaam@yahoo.com (J.Z.); listzhang@163.com (Y.Z.); xuxiaoxia111@scau.edu.cn (X.X.); 2 Laboratory of Insect Microbiology and Biotechnology, Department of Entomology, Faculty of Agricultural Sciences and Technology, Bahauddin Zakariya University, Multan 66000, Pakistan; sfareed@bzu.edu.pk

**Keywords:** Asian tiger, botanicals, dengue, repellency, plant extract, Sophocarpin, Sophordine

## Abstract

In the current study, to combat insecticide resistance, we explored larvicidal, ovicidal, synergistic, and repellent activities of *Sophora alopecuroides* extract and its dominant constituents against *Aedes albopictus*. The results of the toxicity bioassays demonstrated that the extract of *S. alopecuroides* exerted significant larvicidal activity (16.66–86.66%) against the third-instar larvae of *Ae. albopictus* at different concentrations (5–50 ug/mL) and low hatchability of eggs (2.32–75%) at 5–50 ug/mL. The constituents of *S. alopecuroides* showed a synergistic effect when applied as a mixture (LC_30_ + LC_30_) against larvae, while no synergistic effect was observed against the eggs of *Ae. albopictus*. *S. alopecuroides* extract provided 93.11% repellency in the first 90 min and gradually decreased to 53.14% after 240 min, while the positive control DEET (N,N-diethyl-3-methylbenzamide) showed 94.18% in the first 90 min and 55.33% after 240 min. All of the results exhibited a concentration-dependent effect. To the best of our knowledge, this is the first time that a study has identified a highly effective extract of *S. alopecuroides*, which could be used as an alternative agent to control larvae and eggs and to repel adults of *Ae. albopictus*.

## 1. Introduction

Mosquito-transmitted diseases, such as malaria, filariasis, dengue fever, chikungunya, zika, and yellow fever, pose a significant public health concern, as a relatively large proportion of the human population is exposed to these infectious diseases, especially in tropical areas [1,2,3]. Formerly known as a secondary vector, *Aedes albopictus*, in the recent few decades, has emerged as the primary vector in several parts of the world [4]. Though this mosquito is considered the best vector of dengue, it has been reported to also play a major role in the transmission of chikungunya and zika virus [3,5].

In tropical regions, *Ae. albopictus* has effectively adapted itself in suburban and urban environments and also can colonize in different ecological niches [6]. In Southern China, *Ae. albopictus* has been reported as the most important and primary vector of infectious diseases in urban areas [7]. Poor drainage conditions, unsanitary management of solid wastes, and favorable climatic conditions are the main factors that contribute to the increase of mosquito breeding places [8,9]. On the other hand, large-scale use of synthetic insecticides for the control of mosquitoes has led to the development of resistance in populations of insects, non-biodegradability, harmful effects on natural enemies, and environmental pollution [10,11,12,13,14,15,16,17,18]. Despite the fact that insecticides have been used as a major control strategy for mosquitoes, mosquito-transmitted diseases are still prevalent [19]. In the current scenario, there is an imperative need to formulate alternative strategies to control mosquito-transmitted diseases [20].

The use of plant-based products that have insecticidal activity has become the central focus in an effort to combat the development of resistance to insecticides in disease-related insect vectors. In contrast to synthetic insecticides, plant-based products have the features of low toxicity to mammals, easy biodegradability, less or no harm to beneficial insects, and promising control of targeted insects [21,22]. Until now, various kinds of plant-based products have been identified to have potential insecticidal activity; however, pyrethrum, neem, rotenone, and essential oils are considered as the four major types to control insects [23,24].

Plant extracts, produced by plants, are reported to possess high chemical diversity (up to 60 separate components) [25] and have been used for controlling agricultural, household, and medicinal insects [26,27,28]. Due to the diversity of their chemical constituents, the potential biological activity of the different extracts also varies, depending on the origin of plant species, the climatic conditions, and the time of harvesting [29].

Botanicals and botanical components with potential insecticidal activity, due to their rapid degradation, low-cost, and lack of persistence and bioaccumulation in the environment, have been suggested as alternatives to synthetic insecticides for controlling mosquitoes [15,30,31,32].

Recently, the number of studies reporting the toxic effects of plant extracts against mosquitoes has started mounting [33,34,35], as they exhibit potent larvicidal, ovicidal, and repellent activities. Though there are several plant species reported to produce essential oils and plant extracts with high insecticidal activity against mosquitoes, especially against *Ae. albopictus* [36,37,38,39], the activity of *Sophora alopecuroides* extract has not yet been tested. Therefore, the present study aimed to assess, by laboratory bioassays, the larvicidal, the ovicidal, and the repellent activities of *S. alopecuroides* extract and its constituents against *Ae. albopictus*. Furthermore, the efficacy of *S. alopecuroides* extract’s constituents in binary solution form was checked on larvae and eggs to determine their synergistic or antagonistic effects.

## 2. Materials and Methods

### 2.1. Insect Collection and Rearing

Foshan strain *Ae. albopictus* eggs were collected by Professor Dingxing Jiang in 2017. The colony was brought in the Laboratory of Bio-Pesticide Innovation and Application of Guangdong Province, South China Agricultural University, Guangzhou, China, in April 2018. The culture was maintained in a pathogen- and insecticide-free environment at a temperature of 28 ± 2 °C, 60–70% relative humidity, and a 14 h light/10 h dark photoperiod. The eggs were placed in glass beakers (250 mL) for hatching. Newly hatched larvae were shifted into 1000 mL glass jars (15 × 10 cm) and fed on fish food (Godzilla, CST945). Upon 10% pupation, jars were shifted into white cloth cages (30 × 30 cm). Emerged adult males were shifted into separate cages via an electrical aspirator. Male adults were fed on a 10% (*w*/*v*) sugar solution, while females were offered blood meal with white *Albumen* laboratory mice twice a week [40] with ethical approval (SCAU-AEC-2010-04-16). Small beakers (200 mL) with wet filter paper (conical shaped) were used for oviposition.

### 2.2. Plant Materials

The aerial parts of *S. alopecuroides* were collected from the Skardu Baltistan, Pakistan (35°17′25″ N, 75°38′40″ E) in the middle of July 2017. The plant species were identified by comparing the voucher specimen PUP, PH004 (ART004), SK 135, and SK 108 submitted in Herbarium of the University of Peshawar by [41,42].

#### Botanical Formation

*S. alopecuroides* extract (SAE) was prepared by following the method of Wiwattanapatapee [43] using a simple mixing procedure. The semisolid extract was oven-dried and ground into a fine powder. Then, the dried SAE (10 g) was diluted in 10 mL of 70% aqueous methanol and mixed with Tween 80 (30%) and Span 80 (20%) and placed over a magnetic stirrer for 30 min at room temperature; the resultant emulsion contained 10% (*w*/*v*) of SAE. Then, 0.05 g of butylated hydroxytoluene (BHT) was slightly added and mixed to give a homogeneous concentrate mixture. The emulsifiable concentrate was diluted in distilled water for further application. The constituents of *S. alopecuroides* extract used in the present study were determined by our group in a previous study [27].

### 2.3. Larvicidal, Ovicidal, and Repellent Activities

The experiment was carried out according to the directions of the World Health Organization (WHO) protocol [44,45,46] with desired modifications.

#### Larvicidal and Ovicidal Activity of the *S. alopecuroides* Extract and Its Constituents

To assess the larvicidal activity of *S. alopecuroides* extracts against third-instar larvae of *Ae. albopictus*, plastic trays (capacity 250 mL, 125 cm^2^ surface area) were used for the bioassay. Different concentrations (5, 10, 20, 30, and 50 ug/mL) of extract were prepared by diluting 0.01% Tween-80 in 0.5% aqueous acetone. The plastic trays were filled with the required concentration to half of its capacity. The control group only contained aqueous acetone (0.5%) with 0.01% Tween-80. A group of 10 third-instar larvae (F-11) were selected for each treatment. Each treatment was repeated four times. Larvae were transferred to clean water, and 24 h post-treatment, the mortality data were counted up until 5 days [14,47]. Larvae with a lack of movement toward the surface for oxygen intake were counted as dead. The same protocol was used but with different concentrations (20, 40, 60, 80, and 100 ug/mL) for the dominant constituents of *S. alopecuroides* (Sophocarpin and Sophordine) against the third-instar larvae of *Ae. albopictus*

To determine the ovicidal activity of the *S. alopecuroides* extract and its constituents using the same concentrations as mentioned above, small plastic trays (250 mL) were used for the assay. Freshly laid eggs were collected on filter paper. A disinfected blade was used to cut the area of filter paper with 30 eggs. Wet filter papers were air-dried; later, these filter papers with eggs were dipped and placed on a plastic tray containing the relevant treatment (125 mL). Aqueous acetone (0.5%) containing 0.01% Tween-80 was set as control. Each treatment was replicated four times. The date regarding egg hatching was recorded after 24 h of each treatment up until 5 days.

### 2.4. Evaluation of the Synergistic/Antagonistic Relation between the Constituents of S. alopecuroides Extract

Sophocarpin and Sophordine were used in the pre-experimentation against the third-instar larvae and eggs of *Ae. albopictus*; with the help of pre-experimentation data, lethal and sublethal doses were calculated. Sublethal doses (LC_20_ and LC_30_) were individually treated for obtaining the observed mortality of each constituent. Furthermore, these doses were mixed (LC_20_ + LC_20_ and LC_30_ + LC_30_) and treated on the third-instar larvae and eggs of *Ae. albopictus*. Actual mortalities and expected mortalities were compared by using the following formula, where “*E*” represents expected mortality while *O_a_* and *O_b_* refer to the observed mortalities of the compounds in pairs [48]:

$$E=o_{a}+o_{b}\left(1-o_{a}\right)$$

The mixture effect was designated as antagonistic, additive, or synergistic by using the following formula, where *O_m_* is the observed mortality from a binary mixture; *E* is the expected mortality$;\;x^{2}$ represents the Chi value—a pair with an $x^{2}$ value >3.84 and with higher than expected larval mortality is considered as synergistic, while $x^{2}\;$ values <3.84 are taken as antagonistic [49]:

$$x^{2}=\frac{{\left(o_{m}-E\right)}^{2}}{E}\times 100$$

### 2.5. Adult Repellent Activity of the S. alopecuroides Extract

To find out the repellent activity of the *S. alopecuroides* extract, a WHO protocol was followed [50].

#### 2.5.1. Preparation of Adult Females

A group of 240 (60/replication) starved adult females (F-11) were collected for each treatment. Females were kept with males for five days to ensure copulation. To check initial host-seeking behavior, hand spraying with ethanol was used for thirty seconds; if ten females landed for blood-feeding, the experiment was performed—otherwise it was not.

#### 2.5.2. Bioassay

Treatments were performed in cages (30 × 30 cm) made of white cotton cloth with two openings (15 cm diameter) on either side. The extract was formulated as previously described [51], and DEET (N,N-diethyl-3-methylbenzamide) was taken as a positive control. Hands were entirely covered with three layers of latex gloves. An area of 7 cm^2^ was left exposed on the dorsal side. The extract was then applied to the exposed surface. Each treatment was carried out only if, on the control hand, at least 10 females landed for biting. The treated hand was then introduced into the cage containing 60 starved females every 30 min for a period of 1 min. The repellency of extract was assessed from the beginning of the application to 240 min. The time in which two mosquitoes bit the exposed area or one bite was observed in each of the two consecutive exposure periods was registered as Total Protection Time (TPT) [52]. Data were recorded until the end of 240 min. Every time females tried to feed on the hand, it was shaken to avoid the bite but it was counted as a blood meal. The percentage of repellency was calculated by the following equation.

% Repellency (R)=(Ta−Tb/Ta)×100

where R is the percent of repellency, Ta is the number of mosquitoes in the control group, and Tb is the number of mosquitoes in the treated group.

### 2.6. Statistical Analysis

Statistical analysis of the toxicity data was performed using Probit analysis [53]. Mortality was corrected by using the Abbott formulae [54]. Lethal and sub lethal concentrations (LC_50_, LC_30_ and LC_20_) were determined using SPSS 17.0 (SPSS Inc., Chicago, IL, USA) and Polo Pc (Petaluma, CA, USA) The statistical value of *p* < 0.05 were considered as significantly different. Data regarding bioassay were analyzed by Tukey’s test using SPSS 17.0. 

## 3. Results

### 3.1. Larvicidal and Ovicidal Activity of Sophora Alopecuroides Extract and Its Constituents

#### 3.1.1. Larvicidal and Ovicidal Activity of Sophora Alopecuroides Extract

**Figure 1:**
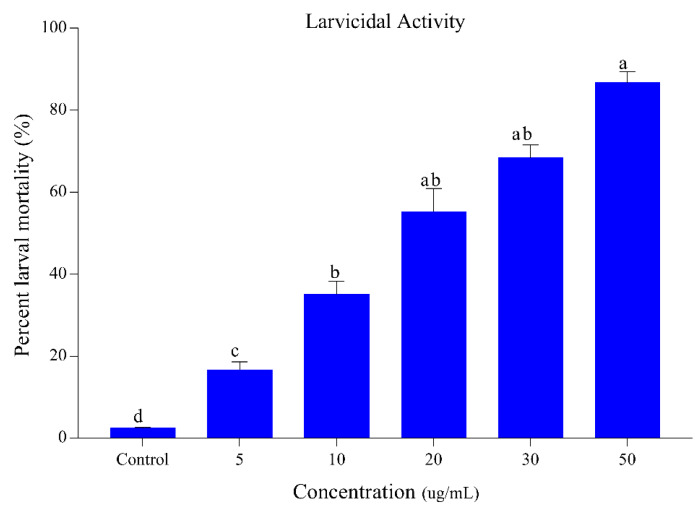
Percentage mortality of *Ae. albopictus* third-instar larvae following *S. alopecuroides* extract treatment at different concentrations. Blue bars represent the mortality of *Ae. albopictus* third-instar larvae after exposure to different concentrations (5, 10, 20, 30, and 50 ug/mL) of *S. alopecuroides* (SACE) extract. Error bars show 95% confidence intervals (CIs). Different letters indicate significant differences at *p* < 0.05.

The results regarding the larvicidal activity of *S. alopecuroides* extract against the third-instar larvae of *Ae. albopictus* are listed in Figure 1. The extract of *S. alopecuroides* displayed the highest mortality ranging between 16.66% and 86.66% at a concentration range of 5–50 ug/mL. Our results demonstrate concentration-dependent larval mortality with a maximum mortality achieved at a concentration of 50 ug/mL. The chi-square values were significant at *p* < 0.05. Lethal (LC_50_) and sub-lethal (LC_30_) concentration (95% CL) values for larvae are presented in Table 1.

**Figure 2:**
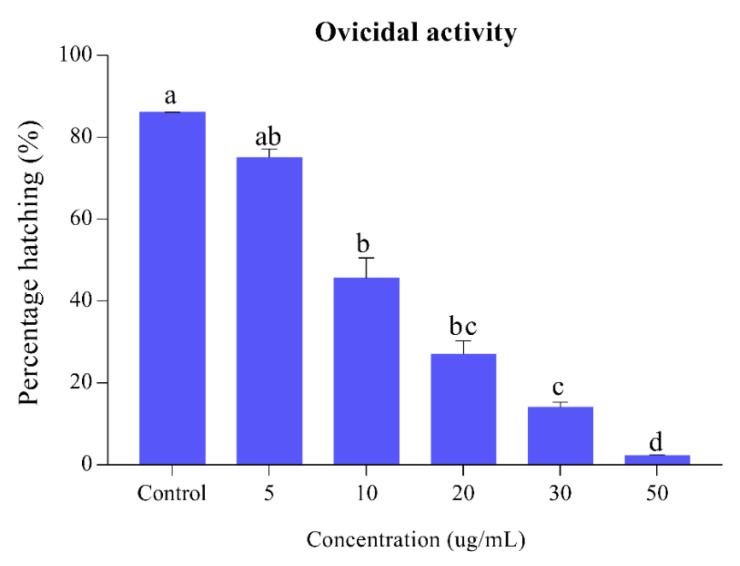
Percentage of *Ae. albopictus* eggs hatching when *S. alopecuroides* extracts treated at different concentrations. Blue bars represent the egg hatching percentage of *Ae. albopictus* third-instar larvae after exposure to different concentrations (5, 10, 20, 30, and 50 ug/mL) of *S. alopecuroides* (SACE) extract. Error bars show 95% confidence intervals (CIs). Different letters indicate significant differences at *p* < 0.05.

The results of the egg hatchability of *Ae. albopictus* against the different concentrations of *S. alopecuroides* extract are presented in Figure 2. Our results of egg hatchability exhibited an inversely proportional relationship to different concentrations of the extract. *S. alopecuroides* provides egg hatchability at different concentrations ranging between 2.32% and 75%, at a concentration range of 5–50 ug/mL. Lethal (LC_50_) and sub-lethal (LC_30_) concentration (95% CL) values for eggs of *Ae. albopictus* are presented in Table 1.

#### 3.1.2. Larvicidal and Ovicidal Activity of Sophocarpin and Sophordine.

**Figure 3:**
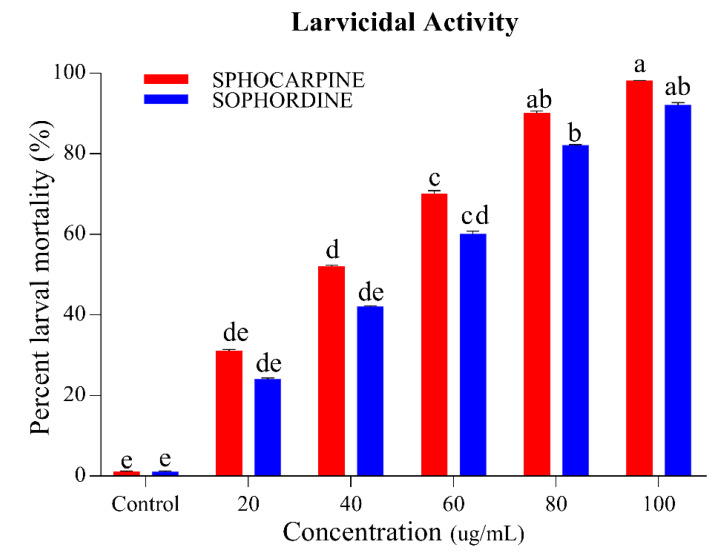
Percentage of larval mortality of *Ae. albopictus* (third-instar larvae) when Sophocarpin and Sophordine were applied at different concentrations (20, 40, 60, 80, and 100 ug/mL). Red bar indicates larval mortality due to Sophocarpin and blue bars represent larval mortality percentage of Sophordine. Error bars show 95% confidence intervals (CIs). Different letters indicate significant differences at *p* < 0.05.

Figure 3 shows the larvicidal activity of the dominant constituents of *S. alopecuroides* extract. Concentration-dependent results were seen. The maximum larval mortality was observed in higher concentrations with a significant value of chi-square (*p* < 0.05); Sphocarpine shows the highest larval mortality between 31% and 98%, followed by Sophordin*e* between 24% and 92% at a concentration limit of 20–100 ug/mL (Figure 3). Using the results of larvicidal activity, lethal (LC_50_) and sub-lethal (LC_30_) concentrations were calculated (Table 2).

**Figure 4:**
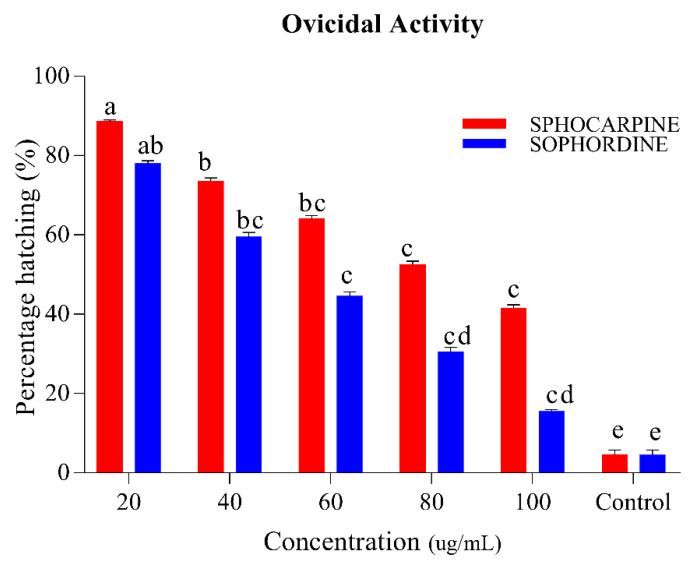
Percentage of *Ae. albopictus* eggs hatching when Sophocarpin and Sophordine were treated at different concentrations (20, 40, 60, 80, and 100 ug/mL). The red bar shows the percent hatching of Sophocarpin, while the blue bars represents the egg hatching percentage of Sophordin of *S. alopecuroides* (SACE) extract. Error bars show 95% confidence intervals (CIs). Different letters indicate significant differences at *p* < 0.05.

The results of ovicidal activity in the eggs of *Ae. albopictus* after treatment of Sophocarpin and Sophordine are shown in Figure 4. An inverse relation between concentration and percentage of hatching was observed in both chemicals; an increase of concentration shows a decrease in the percentage of hatching. Sophocarpin provides egg hatchability ranging between 41.5% and 88.5% at a concentration range of 20–100 ug/mL, while Sophordine shows more significant control than Sophocarpin, because egg hatchability varies from 15.5% to 78% in same concentrations limit (*p* < 0.05). Using the results of larvicidal activity, lethal (LC_50_) and sub-lethal (LC_30_) concentrations were calculated (Table 2).

### 3.2. Evaluation of Synergistic/Antagonistic Relation between the Constituents of S. alopecuroides Extract

Table 3 shows the synergistic/antagonistic relation between the constituents of *S. alopecuroides* extract. A synergistic effect was noticed after the treatment of the mixture containing Sophocarpin and Sophordine (LC_30_ + LC_30_) against the larvae of *Ae. albopictus*. As compared to the expected mortality (42.56%), a significant increase in actual mortality (60 ± 0.40) was noticed upon applying LC_30_ + LC_30_ of Sophocarpin and Sophordine. Meanwhile, a mixture containing LC_20_ + LC_20_ of Sophocarpin and Sophordine showed an antagonistic effect on larval mortality. The ovicidal activity showed no synergistic effect when both mixtures (LC_30_ + LC_30_ and LC_20_ + LC_20_) of Sophocarpin and Sophordine were applied. Actual mortality was lower than expected mortality in both mixtures (LC_30_ + LC_30_ and LC_20_ + LC_20_) of Sophocarpin and Sophordine against the eggs of *Ae. albopictus*.

### 3.3. Adult Repellent Activity of the S. alopecuroides Extract

**Figure 5:**
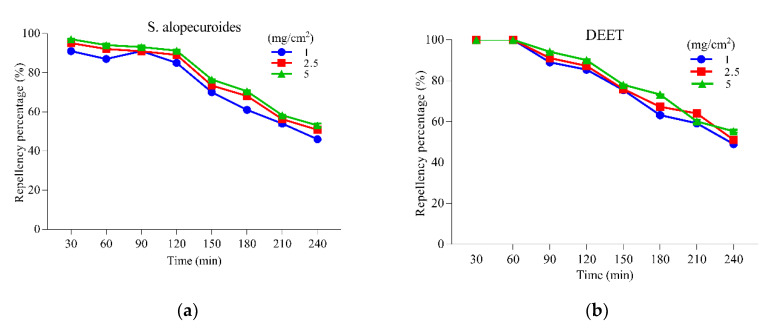
Repellent activities of *Sophora alopecuroides* extract against the adult females of *Ae. albopictus* at different concentrations. (**a**) Repellent activity of *S. alopecuroides* extract against the adult females of *Ae. albopictus* at different concentrations. The blue line represents repellency percentage at 1 mg/cm^2^; the red line represents repellency percentage at 2.5 mg/cm^2^; the green line represents repellency percentage at 5 mg/cm^2^. (**b**) Repellent activity of DEET against the adult females of *Ae. albopictus* at different concentrations. The blue line represents repellency percentage at 1 mg/cm^2^; the red line represents repellency percentage at 2.5 mg/cm^2^; the green line represents repellency percentage at 5 mg/cm^2^.

The results of the repellent activity of *S. alopecuroides* extract, along with positive control (DEET), against the adult female *Ae. albopictus* are presented in Figure 5. Our results displayed a direct relationship between repellent activity and different concentrations of the extract, while an inverse relationship was observed with the time of application. The results of repellent activity demonstrate that *S. alopecuroides* extract resulted in 93.11% protection for 90 min, which kept reducing with time and, at 240 min, only 53.14% protection was provided at a 5 mg/cm^2^ concentration (Figure 5a). However, the positive control (DEET) also provided protection percentage (94.18%) in the first 90 min and 55.33% in 240 min at a 5 mg/cm^2^ concentration (Figure 5b).

## 4. Discussion

Here, in the current study, we demonstrated that *S. alopecuroides* extract, though quite different in chemical composition, provided pronounced larvicidal, ovicidal, and repellent activities against *Ae. albopictus*. The chemical composition analysis previously conducted by our group identified that *S. alopecuroides* is dominated by alkaloids, containing Sophocarpin (33.90%), Sophordine (6.23%), anagyrine, (2.77%), matrine (2.38%), and lupanine (1.68%) as major constituents [27].

The results of our larvicidal activity of *S. alopecuroides* extract and its constituents exhibited high mortality against the third-instar larvae of *Ae. albopictus*, indicating that the tested extract and its constituents can effectively control *Ae. albopictus* at the larval stage. Additionally, *S. alopecuroides* extract was found to be more effective than a single active compound due to the synergism of its active ingredients, which may be effective in managing the resistant population of *Ae. albopictus*. The larval mortality observed in our findings demonstrated a concentration-dependent manner, as larval mortality increased with an increase in the concentration of *S. alopecuroides* extract and its constituents (Sophocarpin and Sophordine), and maximum mortality was achieved at higher concentrations. In previous studies, a similar trend was observed when the essential oils of *Eucalyptus*, *Camaldulensis*, *Eucalyptus nitens*, and *Cinnamomum osmophloeum* were evaluated against *Ae. albopictus*, respectively [55,56,57]. Although our results of different levels of mortality achieved by different plant derivatives are in accordance with other reports, further studies are required to find out the mode of action of the extract and the constituents of *S. alopecuroides* toward the overall toxicity.

The egg hatchability of *Ae. albopictus* was also greatly affected by *S. alopecuroides* extract and its constituents. The egg hatchability results demonstrated an inverse relation with different concentrations of the plant extract, as significantly reduced egg hatchability was observed with an increase in the concentration. Our findings reveal that the extract of *S. alopecuroides* plays a significant role in egg hatch inhibition, as the egg hatchability was significantly lower in those exposed to different concentrations of the *S. alopecuroides* (2.32–75%). When the constituents of *S. alopecuroides* extract were applied against the eggs of *Ae. albopictus*, Sophordine showed more significant control than Sophocarpin. Previously, it was reported that, when the eggs of *Ae. albopictus* were exposed to various concentrations of *Ipomoea cairica* L. leaf extract, a significant decrease in egg hatchability was recorded (0–87%), higher than the one recorded in our findings [58]. The remarkable larvicidal and ovicidal activities of *S. alopecuroides* extract might be due to the presence of a variety of alkaloids that possess potential insecticidal activity. Previously, it was reported that *S. alopecuroides* alkaloids showed high toxicity against aphids [59], *Plagiodera versicoloraetc* [60], *Plutella xylostella*, *Helicoverpa armigera* [61], and *Leucania separate* [62]. Though, in the present study, the mechanism of action of the plant extract from *S. alopecuroides* was not determined, it has been reported that the alkaloids of *S. alopecuroides* are involved in the inhibition of two commonly found enzymes (esterase and carboxylesterase) in insects [63]. The dominant components of the plant extracts also have their own potential against specific insects, but potential varies as compared to the basic extract [64,65,66].

The repellent activity results of *S. alopecuroides* extract provided promising and remarkable repellency potential against the adult females of *Ae. albopictus*; the protection provided by *S. alopecuroides* extract was 93.11% in the first 90 min, which gradually decreased to 53.14% after 240 min, and positive control DEET showed almost the same repellency potential. The repellent activity provided by *S. alopecuroides* extract was also better than previously used repellents against *Ae. albopictus*, such as the *E. nitens* essential oil, and its main component, 1,8-cineole [57].

The good potential of repellency shown by *S. alopecuroides* extract might be attributed to the presence of sesquiterpenes in a high proportion, as sesquiterpenes have been reported to play an active role in repellency against mosquitoes due to their attributes of being less volatile and having a long-lasting effect [67,68,69].

## 5. Conclusions

Concluding our findings, this is the first report investigating the larvicidal, the ovicidal, the synergistic, and the repellent activities of *S. alopecuroides* extract and its constituents. Our results demonstrated that *S. alopecuroides* extract provides promising and remarkable larvicidal and ovicidal activities against *Ae. albopictus* that could be associated with the presence of alkaloids. Either a synergistic or an antagonistic effect of the *S. alopecuroides* constituents against larvae was observed, depending on the applied dose. On the other hand, *S. alopecuroides* was identified as a potent repellent of the adult females of *Ae. albopictus* due to its high potential of repellency. However, further studies are required to determine the contribution of the major and the minor components of *S. alopecuroides* extract toward the mode of action and the repellency.

## Figures and Tables

**Table 1:** Lethal and sub-lethal doses of *S. alopecuroides* extract against *Ae. albopictus* larvae and eggs.

Treatments		LC_50_ (ug/mL)	LC_30_ (ug/mL)	Slope ± SE	*χ*^2^ (*d.f*)	*p*-Value
*Sophora alopecuroides*	Larvicidal activity	4.81 (3.04–6.33)	2.94 (2.47–3.90)	1.99 ± 0.186	1.28 (4)	0.002
	Ovicidal activity	12.08 (10.40–14.28)	4.22 (3.33–5.39)	1.81 ± 0.182	0.38 (4)	0.001

LC_50_, lethal concentration 50% mortality; LC_30_, lethal concentration 30% mortality; χ^2^, chi-square; d.f, degrees of freedom; SE, standard error (95% CL).

**Table 2:** Lethal and sub-lethal doses of Sophocarpin and Sophordine against *Ae. albopictus* larvae and eggs.

Activities	Chemicals	LC_50_ ug/mL	LC_30_ ug/mL	LC_20_ ug/mL	SLOP ± SE	χ^2^ (*d.f*)	*p*-Value
Larvicidal activity	Sophocarpin	33.44 (29.99 ± 36.69)	27.63 (24.20 ± 30.74)	17.73 (14.51 ± 20.65)	3.05 ± 0.28	18.33 (4)	0.002
	Sophordine	41.09 (37.43 ± 44.69)	33.52 (29.92 ± 36.85)	20.89 (17.44 ± 24.03)	2.86 ± 0.27	14.15 (4)	0.005
Ovicidal activity	Sophocarpin	83.82 (76.44 ± 93.68)	62.47 (57.62 ± 68.11)	31.57 (27.39 ± 35.28)	1.98 ± 0.18	4.98 (4)	0.003
	Sophordine	46.69 (43.52 ± 49.91)	36.64 (33.62 ± 39.50)	20.87 (18.01 ± 23.51)	2.40 ± 0.18	14 (4)	0.002

LC_50_, lethal concentration 50% mortality; LC_30_, lethal concentration 30% mortality; χ^2^, chi-square; d.f, degrees of freedom; SE, standard error (95% CL).

**Table 3:** Joint action of the dominant constituents from *S. alopecuroides* (Sophocarpin and Sophordine).

Larvicidal Activity (%)								
			Pure Compound		Binary Mixture			
Compound a	Compound b	Dose (ug/mL)	Observed a	Observed b	Expected	Observed	χ^2^	Effect
Sophocarpin	Sophordine	LC_30_ + LC_30_	24.5	32.8	42.5	60.2	7.14	Synergistic
Sophocarpin	Sophordine	LC_20_ + LC_20_	29.7	33.6	44.1	56.3	3.26	Antagonistic
**Ovicidal Activity** **(** **%)**								
Sophocarpin	Sophordine	LC_30_ + LC_30_	36.5	33.4	44.5	43.1	0.047	Antagonistic
Sophocarpin	Sophordine	LC_20_ + LC_20_	14.5	18.2	27.7	27	0.022	Antagonistic

## References

[1] Gratz N. (2004). Critical review of the vector status of *Aedes albopictus*. Med. Vet. Entomol..

[2] Enserink M. (2008). A mosquito goes global. Science.

[3] Chouin-Carneiro T., Vega-Rua A., Vazeille M., Yebakima A., Girod R., Goindin D., Dupont-Rouzeyrol M., Lourenço-de-Oliveira R., Failloux A.-B. (2016). Differential susceptibilities of *Aedes aegypti* and *Aedes albopictus* from the Americas to Zika virus. PLoS Negl. Trop. Dis..

[4] Rochlin I., Ninivaggi D.V., Hutchinson M.L., Farajollahi A. (2013). Climate change and range expansion of the Asian tiger mosquito (*Aedes albopictus*) in Northeastern USA: Implications for public health practitioners. PLoS ONE.

[5] Vega-Rua A., Zouache K., Caro V., Diancourt L., Delaunay P., Grandadam M., Failloux A.-B. (2013). High efficiency of temperate *Aedes albopictus* to transmit chikungunya and dengue viruses in the Southeast of France. PLoS ONE.

[6] Hanson S.M., Craig G.B. (1994). Cold acclimation, diapause, and geographic origin affect cold hardiness in eggs of *Aedes albopictus* (Diptera: Culicidae). J. Med. Entomol..

[7] Wu J.Y., Lun Z.R., James A.A., Chen X.G. (2010). Dengue fever in mainland China. Am. J. Trop. Med. Hyg..

[8] Medlock J.M., Hansford K.M., Schaffner F., Versteirt V., Hendrickx G., Zeller H., Bortel W.V. (2012). A review of the invasive mosquitoes in Europe: Ecology, public health risks, and control options. Vector-Borne Zoonotic Dis..

[9] Shoukat R.F., Hassan B., Shakeel M., Zafar J., Li S., Freed S., Xu X., Jin F. (2019). Pathogenicity and Transgenerational Effects of Metarhizium anisopliae on the Demographic Parameters of *Aedes albopictus* (Culicidae: Diptera). J. Med. Entomol..

[10] Shakeel M., Farooq M., Nasim W., Akram W., Khan F.Z.A., Jaleel W., Zhu X., Yin H., Li S., Fahad S. (2017). Environment polluting conventional chemical control compared to an environmentally friendly IPM approach for control of diamondback moth, *Plutella xylostella* (L.), in China: A review. Environ. Sci. Pollut. Res..

[11] Ocampo C.B., Salazar-Terreros M.J., Mina N.J., McAllister J., Brogdon W. (2011). Insecticide resistance status of *Aedes aegypti* in 10 localities in Colombia. Acta Trop..

[12] Miranda J.E., Navickiene H.M.D., Nogueira-Couto R.H., De Bortoli S.A., Kato M.J., da Silva Bolzani V., Furlan M. (2003). Susceptibility of *Apis mellifera* (Hymenoptera: Apidae) to pellitorine, an amide isolated from Piper tuberculatum (Piperaceae). Apidologie.

[13] Lin C.-Y., Wu D.-C., Yu J.-Z., Chen B.-H., Wang C.-L., Ko W.-H. (2009). Control of silverleaf whitefly, cotton aphid and kanzawa spider mite with oil and extracts from seeds of sugar apple. Neotrop. Entomol..

[14] Shoukat R.F., Freed S., Ahmad K.W., Rehman A.-U. (2018). Assessment of Binary Mixtures of Entomopathogenic Fungi and Chemical Insecticides on Biological Parameters of *Culex pipiens* (Diptera: Culicidae) under Laboratory and Field Conditions. Pak. J. Zool..

[15] Malik S.U., Zia K., Ajmal M., Shoukat R.F., Li S., Saeed M., Zafar J., Shoukat R.F. (2018). Comparative efficacy of different insecticides and estimation of yield losses on BT and non-BT cotton for thrips, red cotton bug, and dusky cotton bug. J. Entomol. Zool. Stud..

[16] Zafar J., Freed S., Khan B.A., Farooq M. (2016). Effectiveness of Beauveria bassiana Against Cotton Whitefly, *Bemisia tabaci* (Gennadius) (Aleyrodidae: Homoptera) on Different Host Plants. Pak. J. Zool..

[17] Ahmed K., Freed S., Shoukat R.F., Ahmad K.W. (2020). Efficacy of Entomopathogenic Fungi with Insecticides Mixtures against *Oxycarenus hyalinipennis* (Costa) (Lygaeidae: Hemiptera). Pak. J. Zool.

[18] Ahmed S.M., Saeed M., Nawaz A., Usman M., Shoukat R.F., Li S., Zhang Y., Zeng L., Zafar J., Akash A. (2018). Monitoring of quantitative and qualitative losses by lepidopteran, and homopteran pests in different crop production systems of *Brassica oleracea* L.. J. Entomol. Zool. Stud..

[19] Enayati A.A., Hemingway J. (2006). Pyrethroid insecticide resistance and treated bednets efficacy in malaria control. Pest. Biochem. Physiol..

[20] Shoukat R.F., Zafar J., Shakeel M., Zhang Y., Freed S., Xu X., Jin F. (2020). Assessment of Lethal, Sublethal, and Transgenerational Effects of Beauveria Bassiana on the Demography of *Aedes albopictus* (Culicidae: Diptera). Insects.

[21] Mdoe F.P., Cheng S.-S., Lyaruu L., Nkwengulila G., Chang S.-T., Kweka E.J. (2014). Larvicidal efficacy of *Cryptomeria japonica* leaf essential oils against Anopheles gambiae. Parasite Vector.

[22] Shoukat R.F., Freed S., Ahmad K.W. (2016). Evaluation of binary mixtures of entomogenous fungus and botanicals on biological parameters of *Culex pipiens* (Diptera: Culicidae) under laboratory and field conditions. Int. J. Mosq. Res..

[23] Park Y.-L., Tak J.-H. (2016). Essential oils for arthropod pest management in agricultural production systems. Essential Oils in Food Preservation. Flavor and Safety.

[24] Khan B.A., Freed S., Zafar J., Farooq M., Shoukat R.F., Ahmad K.W., Li S., Zhang Y., Hua Y., Shoukat R.F. (2018). Efficacy of different entomopathogenic fungi on biological parameters of pulse beetle *Callosobruchus chinensis* L. (Coleoptera: Bruchidae). J. Entomol. Zool. Stud..

[25] Bakkali F., Averbeck S., Averbeck D., Idaomar M. (2008). Biological effects of essential oils–A review. Food Chem. Toxicol..

[26] Rizvi S.A.H., Tao L., Zeng X. (2018). Chemical composition of essential oil obtained from *Artemesia absinthium* L. grown under the climatic condition of Skardu Baltistan of Pakistan. Pak. J. Bot..

[27] Rizvi S.A.H., Ling S., Tian F., Liu J., Zeng X. (2018). Interference mechanism of *Sophora alopecuroides* L. alkaloids extract on host finding and selection of the Asian citrus psyllid *Diaphorina citri* Kuwayama (Hemiptera: Psyllidae). Environ. Sci. Pollut. Res..

[28] Rizvi S.A.H., Siquan L., Fajun T., Feng X., Xinnian Z. (2018). Toxicity and enzyme inhibition activities of the essential oil and dominant constituents derived from *Artemisia absinthium* L. against adult Asian citrus psyllid *Diaphorina citri* Kuwayama (Hemiptera: Psyllidae). Ind. Crop. Prod..

[29] Joshi R.K. (2013). Volatile composition and antimicrobial activity of the essential oil of *Artemisia absinthium* growing in Western Ghats region of North West Karnataka, India. Pharm. Biol..

[30] Sukumar K., Perich M.J., Boobar L.R. (1991). Botanical derivatives in mosquito control: A review. J. Am. Mosq. Control Assoc..

[31] Saeed M., Shoukat R.F., Zafar J. (2017). Population dynamics of natural enemies and insect pest in different *Brassica oleracea* (cabbage) growing seasons with different production systems. J. Entomol. Zool. Stud..

[32] Su T., Mulla M.S. (1999). Oviposition bioassay responses of *Culex tarsalis* and *Culex quinquefasciatus* to neem products containing azadirachtin. Entomol. Exp. Appl..

[33] Kumar S., Mishra M., Wahab N., Warikoo R. (2014). Larvicidal, repellent, and irritant potential of the seed-derived essential oil of *Apium graveolens* against dengue vector, *Aedes aegypti* L. (Diptera: Culicidae). Front. Public Health.

[34] Thomas A., Mazigo H.D., Manjurano A., Morona D., Kweka E.J. (2017). Evaluation of active ingredients and larvicidal activity of clove and cinnamon essential oils against *Anopheles gambiae* (sensu lato). Parasite Vector.

[35] Andrade-Ochoa S., Sánchez-Aldana D., Chacón-Vargas K.F., Rivera-Chavira B.E., Sánchez-Torres L.E., Camacho A.D., Nogueda-Torres B., Nevárez-Moorillón G.V. (2018). Oviposition deterrent and larvicidal and pupaecidal activity of seven essential oils and their major components against *Culex quinquefasciatus* Say (Diptera: Culicidae): Synergism–antagonism effects. Insects.

[36] Conti B., Leonardi M., Pistelli L., Profeti R., Ouerghemmi I., Benelli G. (2013). Larvicidal and repellent activity of essential oils from wild and cultivated *Ruta chalepensis* L. (Rutaceae) against *Aedes albopictus* Skuse (Diptera: Culicidae), an arbovirus vector. Parasitol. Res..

[37] Guidobaldi F., May-Concha I., Guerenstein P. (2014). Morphology and physiology of the olfactory system of blood-feeding insects. J. Physiol. Paris.

[38] Giatropoulos A., Kimbaris A., Michaelakis A., Papachristos D.P., Polissiou M.G., Emmanouel N. (2018). Chemical composition and assessment of larvicidal and repellent capacity of 14 Lamiaceae essential oils against Aedes albopictus. Parasitol. Res..

[39] Giatropoulos A., Pitarokili D., Papaioannou F., Papachristos D.P., Koliopoulos G., Emmanouel N., Tzakou O., Michaelakis A. (2013). Essential oil composition, adult repellency and larvicidal activity of eight Cupressaceae species from Greece against *Aedes albopictus* (Diptera: Culicidae). Parasitol. Res..

[40] Barnard D.R., Xue R.-D. (2004). Laboratory evaluation of mosquito repellents against *Aedes albopictus*, *Culex nigripalpus*, and *Ochlerotatus triseriatus* (Diptera: Culicidae). J. Med. Entomol..

[41] Bano A., Ahmad M., Hadda T.B., Saboor A., Sultana S., Zafar M., Khan M.P.Z., Arshad M., Ashraf M.A. (2014). Quantitative ethnomedicinal study of plants used in the skardu valley at high altitude of Karakoram-Himalayan range, Pakistan. J. Ethnobiol. Ethnomed..

[42] Hayat M.Q., Ashraf M., Khan M.A., Yasmin G., Shaheen N., Jabeen S. (2009). Phylogenetic analysis of *Artemisia* L. (Asteraceae) based on micromorphological traits of pollen grains. Afr. J. Biotechnol..

[43] Wiwattanapatapee R., Sae-Yun A., Petcharat J., Ovatlarnporn C., Itharat A. (2009). Development and evaluation of granule and emulsifiable concentrate formulations containing Derris elliptica extract for crop pest control. J. Agric. Food Chem..

[44] WHO (1993). Increasing the Relevance of Education for Health Professionals: Report of a WHO Study Group on Problem-Solving Education for the Health Professions [Meeting Held in Geneva from 20 to 23 October 1992].

[45] WHO (2006). Informal Consultation on Malaria Elimination: Setting up the WHO Agenda, Tunis, 25–26 February 2006.

[46] WHO Study Group (2006). Malaria Vector Control and Personal Protection.

[47] Ahmad K.W., Freed S., Shoukat R.F. (2017). Efficacy of entomopathogenic fungi and botanicals on development of Musca domestica. J. Entomol. Zool. Stud..

[48] Trisyono A., Whalon M.E. (1999). Toxicity of neem applied alone and in combinations with Bacillus thuringiensis to Colorado potato beetle (Coleoptera: Chrysomelidae). J. Econ. Entomol..

[49] Hummelbrunner L.A., Isman M.B. (2001). Acute, sublethal, antifeedant, and synergistic effects of monoterpenoid essential oil compounds on the tobacco cutworm, *Spodoptera litura* (Lep., Noctuidae). J. Agric. Food Chem..

[50] WHO (2006). Malaria Vector Control and Personal Protection: Report of a WHO Study Group.

[51] Keziah E.A., Nukenine E.N., Danga S.P.Y., Younoussa L., Esimone C.O. (2015). Creams formulated with *Ocimum gratissimum* L. and *Lantana camara* L. crude extracts and fractions as mosquito repellents against *Aedes aegypti* L.(Diptera: Culicidae). J. Insect Sci..

[52] Schreck C., McGovern T. (1989). Repellents and other personal protection strategies against *Aedes albopictus*. J. Am. Mosq. Control Assoc..

[53] Ai C., Norton E.C. (2003). Interaction terms in logit and probit models. Econ. Lett..

[54] Abbott W.S. (1925). Abbott’s formula. J. Econ. Entomol..

[55] Cheng S.-S., Huang C.-G., Chen Y.-J., Yu J.-J., Chen W.-J., Chang S.-T. (2009). Chemical compositions and larvicidal activities of leaf essential oils from two eucalyptus species. 2009, 100, 452–456. Bioresour Technol..

[56] Cheng S.-S., Liu J.-Y., Huang C.-G., Hsui Y.-R., Chen W.-J., Chang S.-T. (2009). Insecticidal activities of leaf essential oils from *Cinnamomum osmophloeum* against three mosquito species. Bioresour. Technol..

[57] Alvarez Costa A., Naspi C.V., Lucia A., Masuh H.M. (2017). Repellent and larvicidal activity of the essential oil from Eucalyptus nitens against *Aedes aegypti* and *Aedes albopictus* (Diptera: Culicidae). J. Med. Entomol..

[58] Ahbirami R., Zuharah W.F., Yahaya Z.S., Dieng H., Thiagaletchumi M., Fadzly N., Ahmad A.H., Bakar S.A. (2014). Oviposition deterring and oviciding potentials of *Ipomoea cairica* L. leaf extract against dengue vectors. Trop. Biomed..

[59] Ma T., Yan H., Shi X., Liu B., Ma Z., Zhang X. (2018). Comprehensive evaluation of effective constituents in total alkaloids from *Sophora alopecuroides* L. and their joint action against aphids by laboratory toxicity and field efficacy. Ind. Crop. Prod..

[60] Ma X.M., Liu X.X., Zhang Q.W., Zhao J.Z., Cai Q.N., Ma Y.A., Chen D.M. (2006). Assessment of cotton aphids, *Aphis gossypii*, and their natural enemies on aphid-resistant and aphid-susceptible wheat varieties in a wheat–cotton relay intercropping system. Entomol. Exp. Appl..

[61] Cai Q., Zhang Q., Cheo M. (2004). Contribution of indole alkaloids to *Sitobion avenae* (F.) resistance in wheat. J. Appl. Entomol..

[62] Cai Q.-N., Han Y., Cao Y.-Z., Hu Y., Zhao X., Bi J.-L. (2009). Detoxification of gramine by the cereal aphid *Sitobion avenae*. J. Chem. Ecol..

[63] Wanchun L., Yunshou L., Liyi M., Shin-Foon C. (1999). Toxicity of cytisine against the mustard aphid *Lipaphis erysimi* Kaltenbach (Homoptera: Aphididae) and its effect on esterases. Pest. Biochem. Physiol..

[64] Pavela R. (2015). Acute toxicity and synergistic and antagonistic effects of the aromatic compounds of some essential oils against *Culex quinquefasciatus* Say larvae. Parasitol. Res..

[65] Pavela R. (2016). History, presence and perspective of using plant extracts as commercial botanical insecticides and farm products for protection against insects–A review. Plant Prot. Sci..

[66] Tak J.H., Jovel E., Isman M.B. (2016). Comparative and synergistic activity of *Rosmarinus officinalis* L. essential oil constituents against the larvae and an ovarian cell line of the cabbage looper, *Trichoplusia ni* (Lepidoptera: Noctuidae). Pest Manag. Sci..

[67] Paluch G., Grodnitzky J., Bartholomay L., Coats J. (2009). Quantitative structure−activity relationship of botanical sesquiterpenes: Spatial and contact repellency to the yellow fever mosquito, *Aedes aegypti*. J. Agric. Food Chem..

[68] Garcia-Domenech R., Garcia-Mujica P., Gil U., Casanova C., Mireilli Beltran J., Galvez J. (2010). Search of QSAR models for natural sesquiterpenes repellent activity against the Yellow Fever mosquito, *Aedes aegypti*. Afinidad.

[69] Rizvi S.A.H., Xie F., Ling S., Zeng X. (2019). Development and evaluation of emulsifiable concentrate formulation containing *Sophora alopecuroides* L. extract for the novel management of Asian citrus psyllid. Environ. Sci. Pollut. Res..

